# Coping strategies of families and their relationships with family quality of life during Covid-19 pandemic

**DOI:** 10.1371/journal.pone.0273721

**Published:** 2022-09-30

**Authors:** Yael Fogel, Yaron Sela, Liat Hen-Herbst

**Affiliations:** 1 Department of Occupational Therapy, School of Health Sciences, University of Ariel, Ariel, Israel; 2 The Research Center for Internet Psychology (CIP), Sammy Ofer School of Communication Reichman University, Herzliya, Israel; Flinders University, AUSTRALIA

## Abstract

The COVID-19 pandemic has brought new challenges to almost every aspect of parents’ and their children’s lives, posing an acute threat to the families’ quality of life (FQOL). This study had two aims: (1) to identify changes in family coping-strategy profiles among parents pre- and during the first COVID-19 lockdown and (2) to analyze interactions between the clusters of coping strategies pre-COVID with FQOL during the first lockdown. A sample of 253 parents (58.5% mothers) of children (3 to 18 yr old) completed the Family Pandemic Oriented Personal Evaluation Scales and the FQOL Scale about their family life pre- and during the COVID-19 lockdown. Four family coping-strategy clusters were found. Differences were found between those clusters pre- and during the first COVID-19 lockdown, with a high percentage of families using the positive appraisal strategy more often. Significant interactions were found between the family coping-strategy clusters pre-COVID-19 and the FQOL factors before and during the pandemic. Most families maintained their FQOL levels during the first lockdown. Close and frequent interactions between family members had relationships with positive emotions and significant effects on well-being. Results showed that positive cognitive appraisal was a protective factor against a significant decrease in FQOL during the first COVID-19 lockdown.

## Introduction

The COVID-19 pandemic has disrupted economic stability, increased stress levels, and changed families’ daily routines worldwide [1, 2]. It has created a unique, ongoing disaster context with severe impacts on daily life, including increased uncertainty and range of stressors; new fears related to contagion, illness, and death; and reduced access to protective factors, with no end date [3]. More specific knowledge about families’ coping strategies in stressful events, specifically the COVID-19 lockdown, might help clinical professionals treat clients more effectively. Ultimately, this knowledge can help mitigate negative effects on health, functioning patterns, and overall well-being [4].

The COVID-19 pandemic transformed the dynamics of family life worldwide, and many parents found the situation difficult [4, 5]. Government regulations on social distancing and school closures resulted in parents and children spending most of their time together at home. Parents needed to develop multitasking strategies to continue working for their employers while caring for their children [6]. The sudden halt in the daily routine presented new challenges for parents and children alike, with no clear end in sight [7]. Research has shown that situations in which parents must fill their children’s time at home generate many surprises, tensions, and conflicts that force a reorganization of daily activities and make everyday life challenging [8]. However, effective coping strategies may help make parents and their children less vulnerable to the negative impacts of the pandemics’ difficulties and stressors [9].

Lazarus and Folkman [10] defined *coping* as cognitive and behavioral strategies to manage external or internal demands caused by stressors. The techniques to deal with stress have been defined as *coping strategies* [11]. Most people possess several coping strategies to deal with stress-inducing situations [12]. Although researchers have identified numerous coping strategies, they found reaching a consensus on their classification challenging [13]. Coping strategies can be clustered around several basic dimensions. These include problem-focused (e.g., planning and decision-making), emotion-focused (e.g., venting feelings or using humor), avoidance (e.g., denial and behavioral disengagement), and social support (e.g., seeking help, advice, or comfort from others coping [14]. Emotion-focused coping strategies, which aim to regulate emotional responses and reactivity to stressors, may be especially beneficial when individuals encounter stressors out of their control. That is, they cannot control the stressor [10, 15]—as in the case of the COVID-19 pandemic—given that individual families cannot objectively change the pandemic’s health, societal, or economic consequences. Furthermore, because the current study deals with *families’* coping strategies, the resiliency model of family adjustment and adaptation also contributes to classifying these strategies. This model focuses on coping strategies within two dimensions: (1) the individual-to-family system (internal), or ways the family handles difficulties and problems among its members, and (2) the family-to-social environment (external), or ways the family handles problems or demands that emerge outside its boundaries but affect the family unit and its members [16].

When dealing with the pandemic’s challenges or crises, parents’ resources, perceptions, and coping strategies may determine parenting effectiveness and behaviors [4]. The literature regarded how coping occurs in families focuses on some critical red lights. For example, Phelps and Sperry [17]) stated that the COVID-19 pandemic might increase the children’s risk of maltreatment and adversity if parents do not have adequate coping strategies and parenting skills. When facing COVID-19 stressors, parents might use different types of coping strategies. Cognitive- or appraisal-focused strategies might help parents change their thinking about COVID-19 and reassess the pandemic’s impacts on their family lives. Using problem-focused coping strategies, parents will find ways to deal with the challenges the pandemic brought to their families [4]. The use of positive coping strategies helps parents build distress tolerance, increase social support, form positive meanings, and take goal-directed and value-driven actions during the pandemic [18].

Researchers and scholars have suggested that examining patterns of coping styles, or coping *profiles*, may be more advantageous than traditional statistical techniques to promote understanding of the ways individuals cope with stress [19, 20]. Lazarus and Folkman [10] emphasized that a particular coping style might be helpful in certain situations but not others. Furthermore, coping strategies may be context-dependent, indicating that both the stressor and the environment in which the stressor is present contribute to the choice of strategies used [21].

Folkman et al. [22] considered coping critical in determining whether a stressful event results in adaptive or maladaptive outcomes. Skinner et al. [23, p. 216] contended, “How people deal with stress can reduce or amplify the effects of adverse life events and conditions not just on emotional distress and short-term functioning, but also long-term, on the development of physical and mental health or disorder.”

Family coping is a bridging concept. It comprises cognitive and behavioral components in which family resources, perceptions, and behavioral responses cooperate to rebalance family functioning [24]. Family stress and coping theories focus not on individual outcomes but on positive family functioning indicators such as *cohesion*, meaning family members’ emotional bonding, and *adaptability* [25]. Managing the stressors’ demands is vital because the outcome can escalate to unmanageable levels, especially during a pandemic [26]. Thus, aspects of the family environment, parenting practices, and parent coping likely influence the children’s post-disaster mental health [27].

Previous studies examined mostly individual coping strategies; only a few used cluster-analytical procedures to create more comprehensive profiles of coping strategies [28–30]. To our knowledge, no previous research used this procedure to identify family subgroups in their coping-strategies profiles before and during a global pandemic. Hence, in this study, we aimed to identify family cases defined by similarities in their coping strategies. With this approach, we empirically identified discrete groups or typologies that share similar coping-strategy patterns and suggested a more holistic view of parenting coping strategies before and during the pandemic. Furthermore, we expected to find that families moved from one cluster to another during the pandemic. We hypothesized this assumption based on the impact of the pandemic on previous coping strategies, such as the inability to use social support due to the quarantines.

Moreover, the current research took a step forward to investigate interactions between the family coping-strategy profile and family quality of life (FQOL). Based on the original quality-of-life literature, we conceptualized FQOL as multidimensional, similar to the quality-of-life concept [31, 32]. Specifically, we used the FQOL definition of "a dynamic sense of well-being of the family, collectively and subjectively defined and informed by its members, in which individual- and family-level needs interact" [33, p. 262]. Improving FQOL can positively affect child and family outcomes [34, 35].

This study aimed to identify changes in families’ coping strategies before and during the pandemic and investigate interactions between pre-pandemic coping profiles and FQOL before and during the pandemic. We hypothesized that: (1) Significant differences would be found between families’ coping strategies pre- and during the first COVID-19 lockdown, specifically that coping strategies of the family-to-social environment (external) type would decrease during the COVID-19 lockdown. (2) Significant differences would be found between families’ coping-strategy clusters pre- and during the first COVID-19 lockdown. We expected to find that families moved from one cluster to another during the first lockdown. (3) Significant interactions would be found between family coping-strategy clusters pre- and during the first COVID-19 lockdown on the FQOL Scale factors (family interaction, parenting, emotional well-being, and physical well-being) during the first lockdown.

## Materials and methods

### Sample

Study participants were recruited through online advertisements, social media, word-of-mouth, and the Midgam Project web panel (MIDGAM is an Israeli company that provides internet research infrastructure, http://www.midgampanel.com/research/en/index.asp). The inclusion criterion was families with children between the ages of 3 and 18 yr.; there were no exclusion criteria. The *family* definition used in this study was adapted from one the Beach Center on Disability [36, p. 2] developed: “People who are closely involved in the day‐to‐day affairs of the household and support each other regularly, whether related by blood, marriage or close personal relationship.” The sample size was calculated using G*Power for detecting medium effects with a statistical power of 95%, an alpha of 0.05, and up to 15 predictors [37].

### Procedure

The Ethics Committee of the Faculty of Welfare and Health Sciences, University of Haifa approved the study (No. AU-HEA-Y20200405). One parent of each family signed an online informed consent form and then received a link to the online questionnaires (via the Qualtrics^xm^ platform). The data were collected from April 30 to May 24, 2020. Within that period (i.e., from March 14 to May 27, 2020), the Israeli government issued a stay-at-home order, limiting travel and work except for essential needs (e.g., obtaining food or medicine) [38].

The data regarding the families’ coping strategies and the FOQL pre- and during the pandemic were collected at the same time point. Participants completed the questionnaires twice—once regarding their family before the pandemic and again during it. That is, at the same time point, they retrospectively assessed their coping strategies and FOQL for before the pandemic and for the time of data collection (during the first lockdown). To minimize inaccurate answering behaviors due to the high cognitive load of retrospective questions [39], questions about the respondents’ experiences referenced a specific anchor point (3 months before the pandemic began). Such an anchoring factor can increase recall accuracy, even for subjective assessments like rating health status or subjective well-being [40]. The final sample included 253 families with children younger than 18 yr old. Each parent who completed the process received an online game developed for all family members to play together.

### Measures

#### Online demographics questionnaire

The online demographic questionnaire developed for the current study included questions related to parents’ age, education level (high school, vocational, or academic), number of children in the family, place and type of residence, and income level.

#### Family Crisis Oriented Personal Evaluation Scales

The Family Crisis Oriented Personal Evaluation Scales (F-COPES) [41] was created to identify families’ problem-solving and behavioral strategies in difficult or problematic situations. The F-COPES draws upon the coping dimensions of the resiliency model of family adjustment and adaptation, which integrates the factors of pile-up, family resources, and meaning/perception.

Cronbach’s alphas were computed for each factor separately and for the total scale and range (.86–.87). The Cronbach’s alphas for the total scale, both before and during the pandemic, were .76 separately.

We used the Hebrew version of this scale (published by Lavee et al. [42, 43]) in this study. This F-COPES includes 27 items in five scales (items are listed in S1 Table):

- Acquiring social support. Seven items (1, 2, 5, 8, 14, 18, 26) measure a family’s ability to actively engage in acquiring support from relatives, friends, neighbors, and extended family. The Cronbach’s alpha before COVID-19 was .80, and during the pandemic was .70.
- Reframing. Seven items (3, 7, 10, 13, 17, 20, 22) assess the family’s capability to redefine stressful events to make them more manageable. The Cronbach’s alpha before COVID-19 was .81, and during the pandemic was .85.
- Seeking spiritual support. Four items (12, 21, 24, 27) focus on the family’s ability to acquire spiritual support. The Cronbach’s alpha before COVID-19 was .71, and during the pandemic was .56.
- Mobilizing family to acquire and accept help. Four items (4, 6, 9, 19) measure the family’s ability to seek out community resources and accept others’ help. The Cronbach’s alpha before COVID-19 was .66, and during the pandemic was .63.
- Passive appraisal. Four items (11, 15, 23, 25) assess the family’s ability to accept problematic issues, minimizing reactivity. These four items must be reversed when scoring. The Cronbach’s alpha before COVID-19 was .67, and during the pandemic was .58.

#### FQOL scale

The FQOL Scale [44] assesses parent perception regarding aspects of FQOL and various facets of functioning and cohesion in families with and without disabled family members [33, 45]. The instrument contains 25 items divided into five subscales. However, in the version used in this study, four questions related to disability-related support were removed because they would not necessarily apply to all participants. Thus, the questionnaire used contained 21 items divided into four subscales. The validity and reliability of this modified FQOL Scale have been confirmed in studies of families without disabilities [33].

Participants indicate their satisfaction regarding each item on a 5-point Likert scale from 1 (*very unsatisfied*) to 5 (*very satisfied*), with higher ratings indicating higher satisfaction. Cronbach’s alphas for each subscale before and during the COVID-19 pandemic, respectively, were .89 and .91 for *family interaction* (Items 1, 7, 10–12, 18); .82 and .86 for *parenting* (Items 2, 5, 8, 14, 17, 19); .75 and .80 for *emotional well-being* (Items 3, 4, 9, 13); and .80 and .81 for *physical well-being* (Items 6, 15, 16, 20, 21).

### Data analysis

The data were analyzed using SPSS version 25. Descriptive statistics for the demographic characteristics were performed using standard deviations and ranges for the continuous variables and frequencies for the discrete variables. Differences in the F-COPES scores pre- and during the COVID-19 pandemic were analyzed with paired *t* tests for the five scales.

To identify and classify naturally occurring coping-strategy patterns, we transformed the scores into standardized *z* scores (*M* = 0; *SD* = 1) and identified multivariate outliers [46], then performed cluster analysis on the four F-COPES strategies. Based on the significant-high correlations between the participants’ *religious observance* from the demographic questionnaire and *seeking spiritual support* levels from the F-COPES (before: *r* = .60, *p* < .001; during: *r* = .51, *p* < .001), we excluded the *seeking spiritual support* scale from the cluster analysis. We then used *k*-means cluster analysis to verify the results. A multivariate analysis of variance (MANOVA) and potshot multiple comparisons revealed that the two-cluster solution produced a significant difference between all cluster groups on each F-COPES subscale. The two-cluster solution’s stability was then tested using a two-thirds random sample to recluster the data [47]. The cluster analyses of the two time points (pre- and during the pandemic) yielded two cluster sets. Hence, we conducted a chi-square test to assess the change in coping-strategy clusters between the two time points.

We conducted repeated-measures MANOVA to analyze differences between the clusters pre- and during the COVID-19 pandemic. Partial eta-squared (η_p_^2^) was reported as effect size, with η_p_^2^ < .04 considered the minimum representing clinically significant, .04 < η_p_^2^ < .25 moderate, and η_p_^2^ > .25 strong effect sizes [48]. *T* tests were conducted to assess differences between groups and components, and Cohen’s *d* [49] was calculated for effect size (.10 was considered a small, .30 medium, and .50 large effect).

## Results

The mean age of parents who completed the questionnaires was 37.49 yr (*SD* = 6.88), and of the second parent was 37.81 yr (*SD* = 7.13); 58.5% of the sample were mothers. The average number of children per family was 2.90 (*SD* = 1.12). Table 1 presents additional demographic characteristics.

**Table 1:** Means, standard deviations, ranges, and frequencies for the sample’s demographic characteristics.

Characteristic	*n* (%)	*M* (*SD*)	Range
Age			
Parent 1 (respondent)		37.49 (6.88)	25–55
Parent 2		37.81 (7.13)	24–55
Level of education Parent 1 (respondent)			
High school	45 (17.8)		
Vocational	25 (9.9)		
Academic	183 (72.3)		
Number of children		2.90 (1.12)	1–9
1	5 (2.0)		
2	104 (41.1)		
3	83 (32.8)		
4 or more	61 (24.1)		
Place of residence			
City	185 (73.1)		
Other	68 (26.9)		
Type of residence			
Private house with garden	86 (34.0)		
Private house without garden	14 (5.5)		
Building with balcony	102 (40.3)		
Building without balcony	51 (20.2)		
Level of income (NIS)^a^			
Below the average monthly salary	160 (63.2)		
Above the average monthly salary	93 (36.8)		

*Note*. *N* = 253.

^a^Average monthly salary according to the Central Bureau of Statistics. 3.43 Israeli new shekels (NIS) equal approximately US$1.00.

### F-COPES differences pre- and during the COVID-19 pandemic

**Fig 1:**
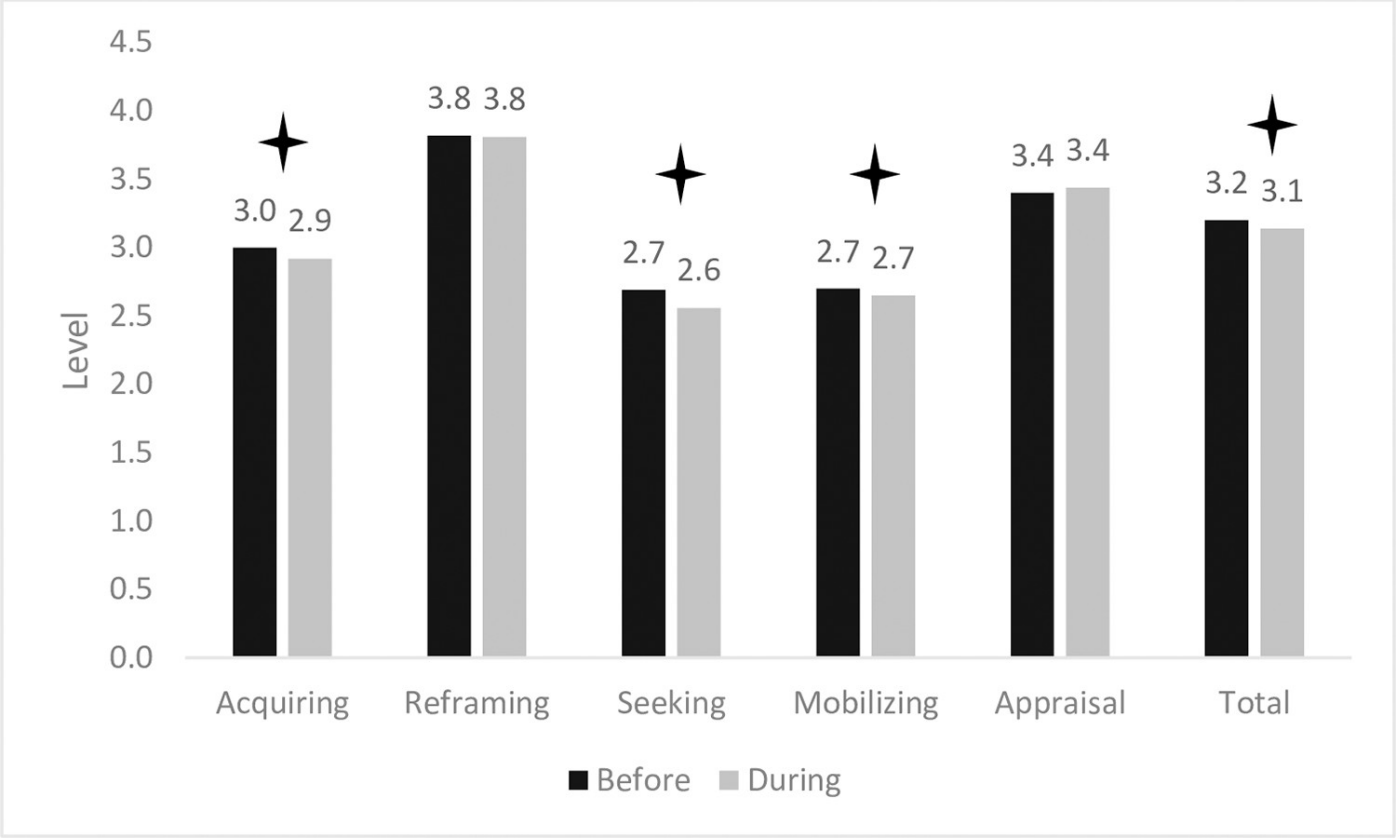
Differences in family pandemic-oriented personal evaluation scale factors pre- and during the COVID-19 pandemic.

Fig 1 illustrates the differences in the F-COPES factors before and during the COVID-19 pandemic (detailed in S2 Table). Results showed that some family abilities decreased during the pandemic compared with beforehand, specifically, the family’s ability to actively acquire support from relatives (before: *M* = 3.00, *SD* = 0.72; during: *M* = 2.92, *SD* = 0.67), *t*(252) = 2.67, *p* < .01; acquire spiritual support (before: *M* = 2.69, *SD* = 1.08; during: *M* = 2.56, *SD* = 0.96), *t*(252) = 3.05, *p* < .01; and seek out community resources and accept help from others (before: *M* = 3.00, *SD* = 0.72; during: *M* = 2.92, *SD* = 0.67), *t*(252) = 2.67, *p* < .01. Further, the total F-COPES level also decreased during the pandemic (before: *M* = 3.20, *SD* = 0.43; during: *M* = 3.14, *SD* = 0.44), *t*(252) = 2.45, *p* = .01.

### Family coping strategies: Cluster analysis

We established the families’ coping-strategy profiles using the scales of the F-COPES questionnaire before COVID-19, assuming the profiles resulted from occurrence data. We found four clusters (Fig 1) built on combinations of the four coping strategies before COVID-19: Cluster 1, consisting of 77 (30.4%) families, represented those using more reframing and appraisal strategies and fewer acquiring and mobilizing strategies. Cluster 2 comprised 86 (34%) families characterized as using all kinds of strategies, including reframing, appraisal, acquiring, and mobilizing. Cluster 3 consisted of 17 (6.7%) families characterized by using a high level of reframing, acquiring, and mobilizing strategies and fewer appraisal strategies. Cluster 4 consisted of 73 (28.9%) families with low use of reframing, acquiring, mobilizing, and appraisal strategies. There were significant differences between the clusters in acquiring, *F*(3, 249) = 74.28, *p* < .001, η^2^ = .47; reframing, *F*(3, 249) = 118.56, *p* < .001, η^2^ = .59; mobilizing, *F*(3, 249) = 176.70, *p* < .001, η^2^ = .68; and appraisal, *F*(3, 249) = 64.47, *p* < .001, η^2^ = .44.

We conducted similar cluster analyses for strategies used during COVID-19 and compared the four new clusters. Cluster 1, consisting of 78 (30.8%) families, represented families with more appraisal but fewer mobilizing strategies. Cluster 2 comprised 87 (34.4%) families characterized as using more appraisal but fewer reframing and mobilizing strategies. Cluster 3 comprised 68 (26.9%) families characterized by using higher levels of appraisal but fewer reframing strategies. Cluster 4 consisted of 20 (7.9%) families characterized by high use of all strategies except appraisal. There were significant differences between the clusters in acquiring, *F*(3, 249) = 53.72, *p* < .001, η^2^ = .39; reframing, *F*(3, 249) = 139.05, *p* < .001, η^2^ = .62; mobilizing, *F*(3, 249) = 124.19, *p* < .001, η^2^ = .59; and appraisal, *F*(3, 249) = 63.40, *p* < .001, η^2^ = .43.

### Comparisons between clusters before and during the COVID-19 pandemic

Table 2 presents the change in clusters between the two time points (before and during the COVID-19 pandemic). A significant change was found between time points, X^2^ = 338.82, *p* < .001. Results showed that most (88.3%) families characterized by using high reframing levels but lower levels of mobilizing strategies before the pandemic (pre-COVID Cluster 1) demonstrated similar strategy use during the pandemic but also used high levels of appraisal (during-COVID Cluster A). However, most (75.6%) families that used similar levels of all strategies before the pandemic (pre-COVID Cluster 2) used mostly appraisal strategies during the pandemic, with low mobilizing levels (during-COVID Cluster A).

**Table 2:** Comparisons between clusters before and during the COVID-19 pandemic.

Pre-COVID During COVID	(1) High reframing; low mobilizing	(2) Similar use (no dominant strategy)	(3) High reframing/ mobilizing; low appraisal	(4) High appraisal; low acquiring/ mobilizing
(A) High appraisal; low mobilizing	7 (9.1%)	65 (75.6%)	1 (5.9%)	5 (6.8%)
(B) High appraisal and reframing; low mobilizing	68 (88.3%)	9 (10.5%)	1 (5.9%)	9 (12.3%)
(C) High appraisal; low reframing	1 (1.3%)	6 (7.0%)	5 (29.4%)	56 (76.7%)
(D) High acquiring, reframing, and mobilizing; low appraisal	1 (1.3%)	6 (7.0%)	10 (58.8%)	3 (4.1%)

*Note*. *N =* 253. Pre-COVID-19 strategies are numbered (1–4); during-COVID-19 strategies are lettered (A–D).

Most (58.8%) families characterized by using high levels of reframing and mobilizing but low appraisal levels before the pandemic (pre-COVID Cluster 3) demonstrated similar strategy use during the pandemic but also used high acquiring levels (during-COVID Cluster D).

Finally, most (76.7%) families that used high levels of reframing and appraisal and low levels of acquiring and mobilizing strategies before the pandemic (pre-COVID Cluster 4) hardly used reframing during the pandemic (during-COVID Cluster C).

### Interactions between coping strategies of family clusters before and FQOL factors during the pandemic

**Fig 2:**
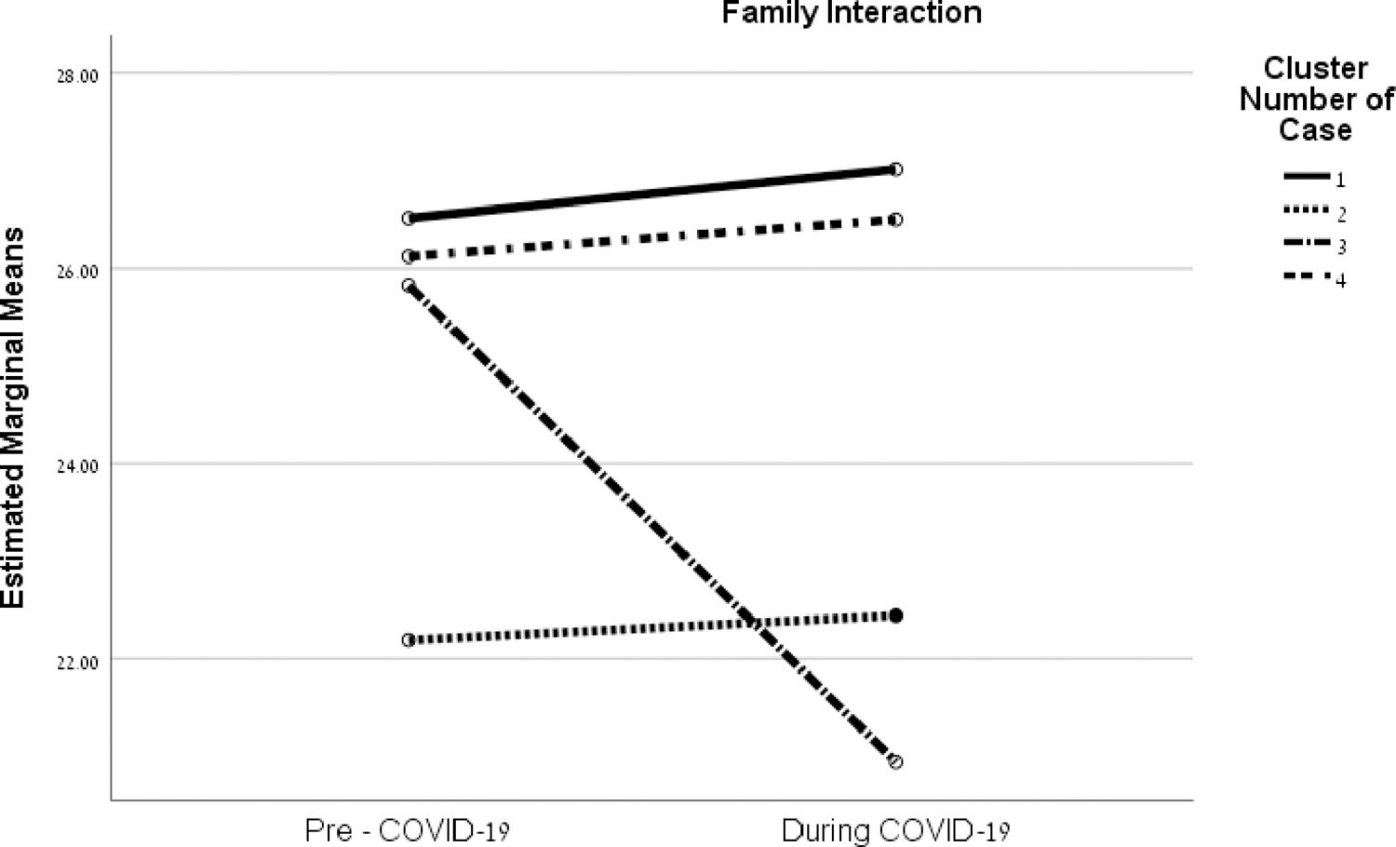
Interactions between coping strategies of families and before/during the COVID-19 crisis on family quality of life factors.

Results showed significant interactions between families’ coping-strategy clusters and time for family interaction, parenting, and emotional well-being. For family interaction, *F*(3, 249) = 18.41, *p* < .001, η^2^ = .18, results showed a significant negative change (decrease) in the pre-COVID Cluster 3 (high reframing and mobilizing; low appraisal) from the time before the pandemic (*M* = 25.82, *SD* = 5.00) to the period during the pandemic (*M* = 20.94, *SD* = 8.78), *t*(76) = 2.96, *p* = .009. Families from the other three clusters showed no significant changes from before to during COVID-19 (Fig 2).

**Fig 3:**
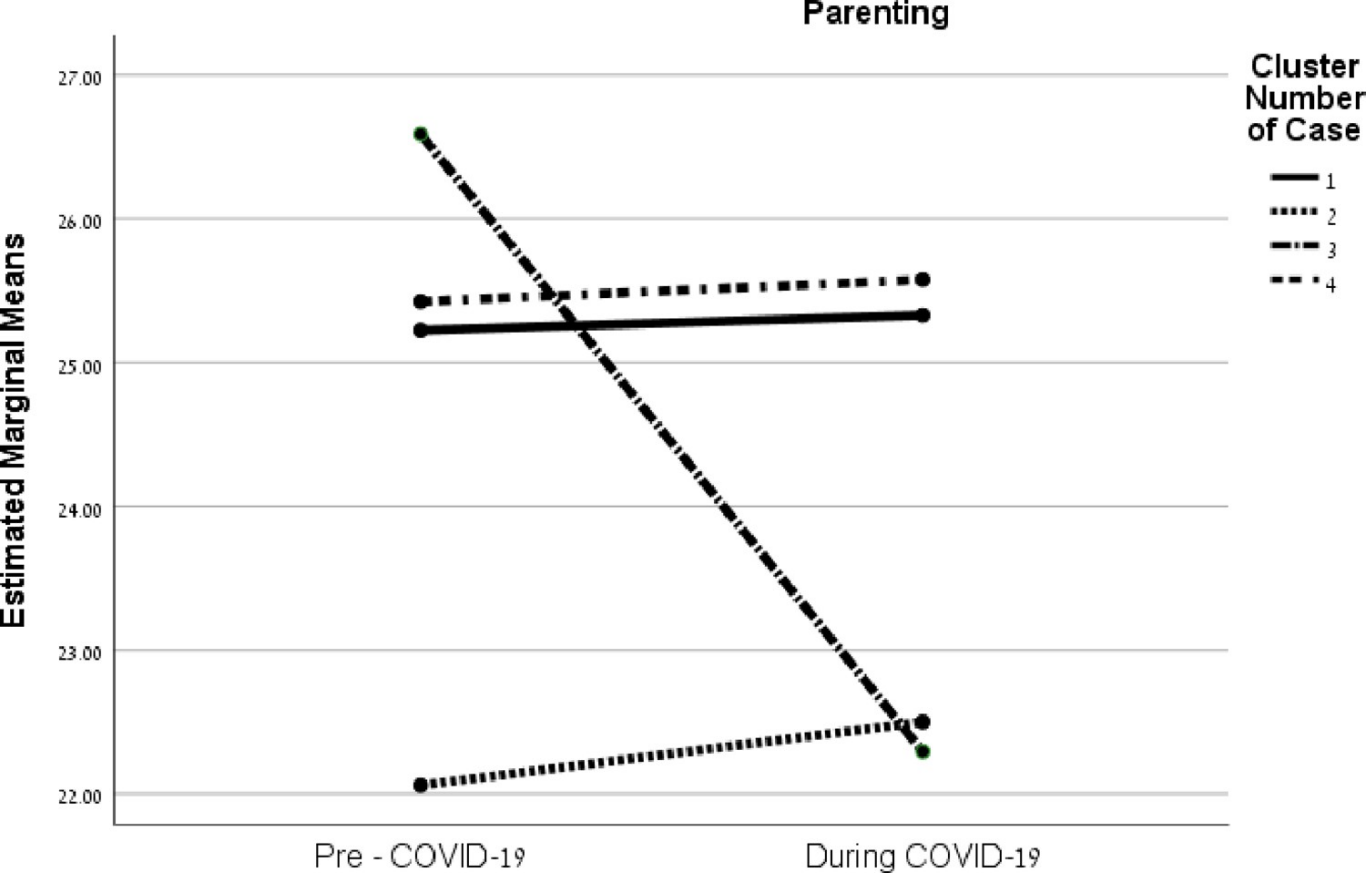
Interactions between coping strategies of families and before/during the COVID-19 crisis on family quality of life factors.

In parenting, *F*(3, 249) = 17.74, *p* < .001, η^2^ = .18, results showed no significant differences in pre-COVID Clusters 1 (high reframing; low mobilizing), 2 (similar use), or 4 (high appraisal; low acquiring and mobilizing). However, Cluster 3 (high reframing and mobilizing; low appraisal) showed a significant negative change (decrease) between the time before (*M* = 26.59, *SD* = 3.02) and the period during the pandemic (*M* = 22.29, *SD* = 8.04), *t*(76) = 2.57, *p* = .02 (Fig 3).

**Fig 4:**
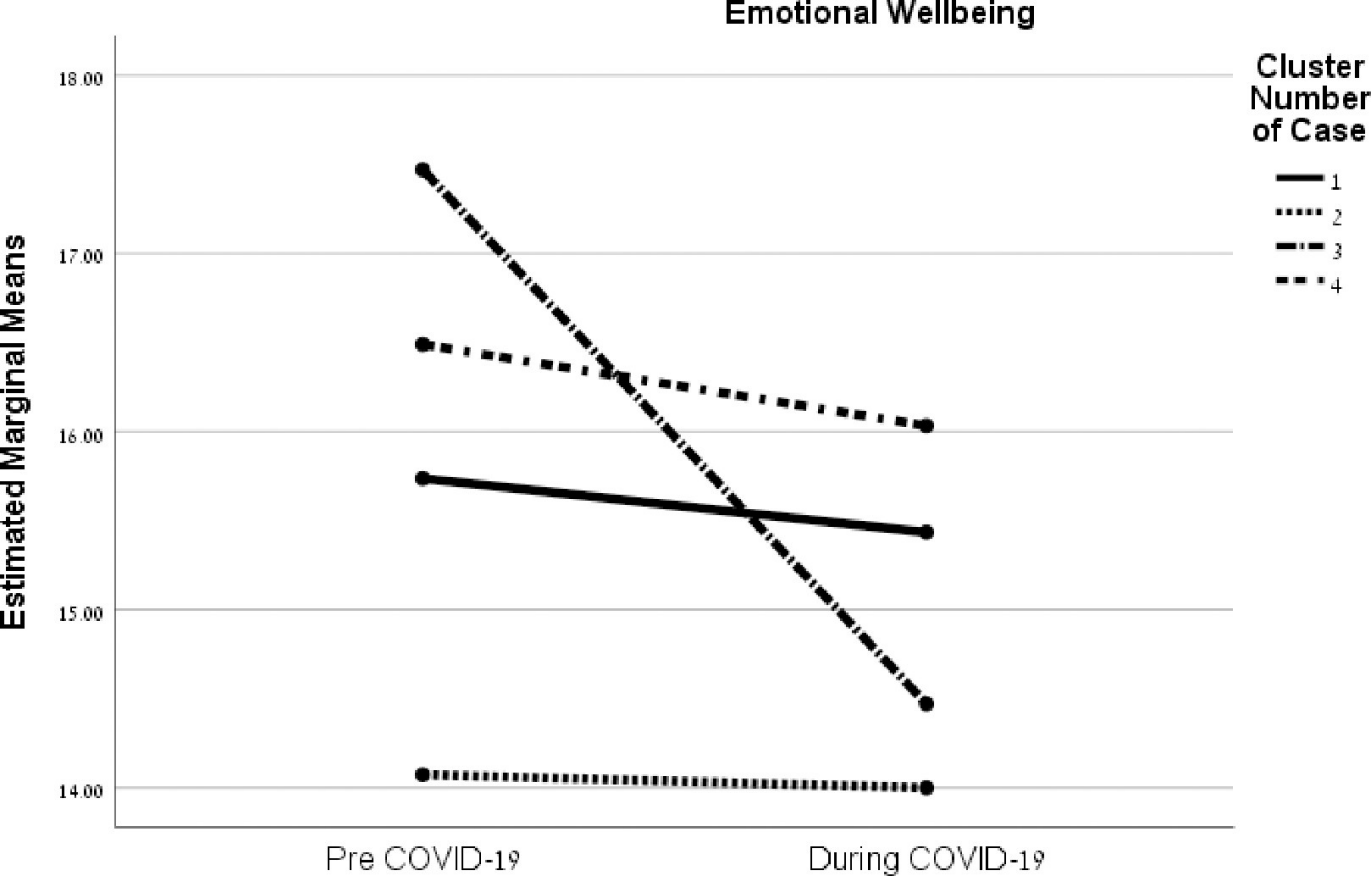
Interactions between coping strategies of families and before/during the COVID-19 crisis on family quality of life factors.

The emotional well-being factor results, *F*(3, 249) = 9.58, *p* < .001, η^2^ = .10), showed that pre-COVID Cluster 2 (similar use, no dominant strategy) had a significant negative (decrease) change between the time before (*M* = 16.37, *SD* = 2.95) and the period during the pandemic (*M* = 15.92, *SD* = 3.51), *t*(76) = 2.09, *p* = .04. Pre-COVID Cluster 3 (high reframing and mobilizing; low appraisal) had a significant negative (decrease) change from before (*M* = 17.47, *SD* = 2.37) to during COVID-19 (*M* = 14.47, *SD* = 5.41), *t*(76) = 2.82, *p* = .01 (Fig 4).

**Fig 5:**
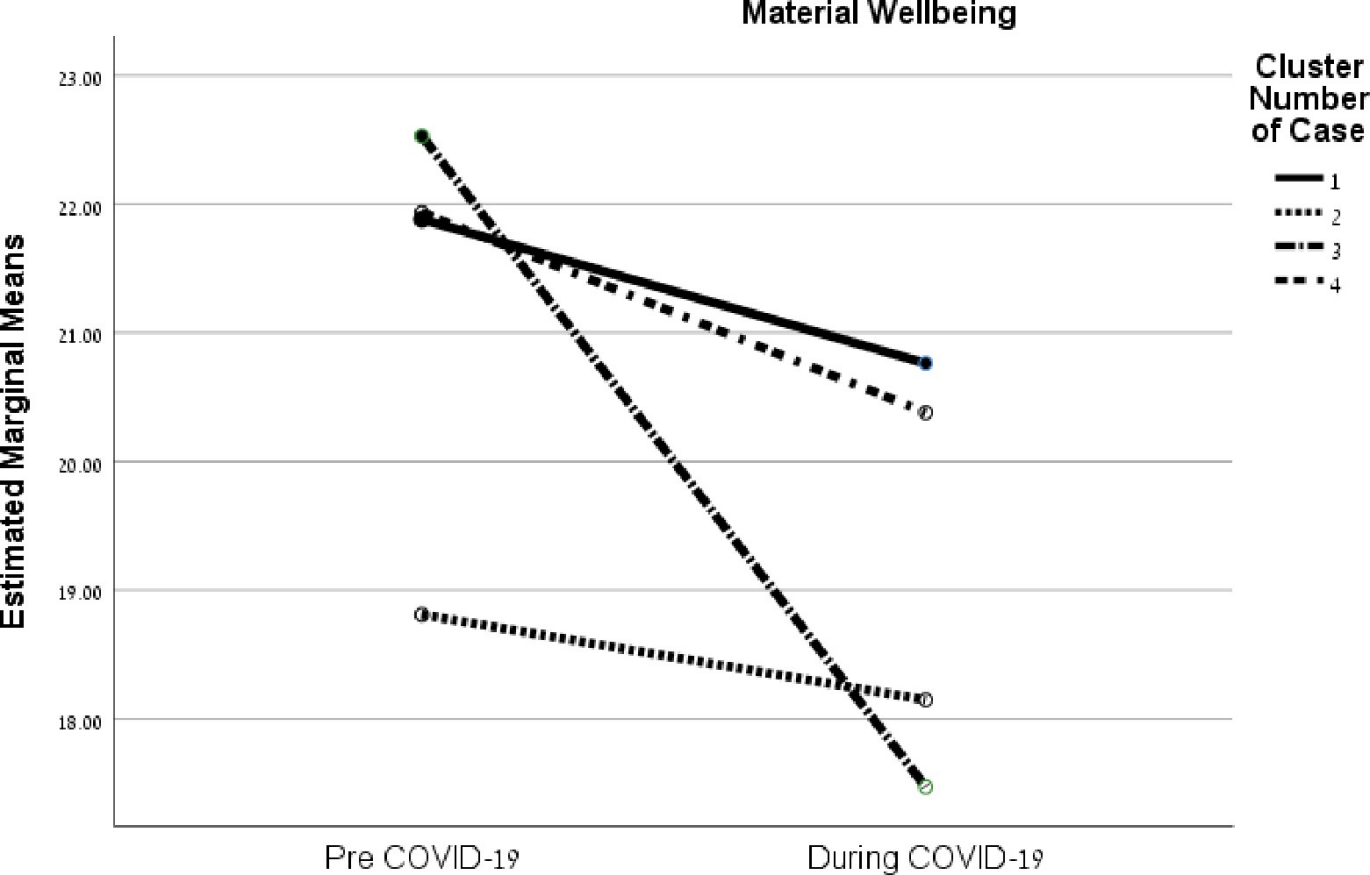
Interactions between coping strategies of families and before/during the COVID-19 crisis on family quality of life factors.

The interactions between these strategy clusters before COVID-19 and the period during the pandemic for the FQOL factors are illustrated in Fig 2 (family interaction), Fig 3 (parenting), Fig 4 (emotional well-being), and Fig 5 (material well-being). (See additional details in S3 Table).

## Discussion

This study aimed to identify changes in families’ coping strategies before and during the COVID-19 pandemic and investigate interactions between pre-pandemic coping profiles and FQOL before and during the pandemic. Previous research showed wide variances in how individuals cope over time with disasters and potentially traumatic events (e.g., [50, 51]). We found that some family abilities were higher before than during the pandemic; specifically, the family’s ability to actively acquire support from relatives, acquire spiritual support, seek out community resources, and accept help from others.

In the terminology of the resiliency model of family adjustment and adaptation [16], parents used significantly fewer external (family-to-social environment) strategies. These findings are predictable under conditions dictated by policymakers, who imposed social distancing to minimize the spread of the virus. These decrees inevitably interfered with crucial social connections and possibly prevented family members from accessing external sources of social and spiritual support that might have helped mitigate distress (e.g., [52, 53]). Furthermore, these findings support distinguishing between coping styles and coping processes. According to Lazarus and Folkman’s [10] framework, *coping style*s are traits suggesting inherent personality characteristics (clusters before the pandemic), whereas *coping processes* exhibit the dynamic interplay of person—or in our case, family—and environment and might explain the changes in coping profiles during the pandemic.

Our factor analysis revealed four family-strategy clusters both before and during the COVID-19 crisis, but the clusters changed between the two periods. We looked deeply at the differences between the four clusters before and during the pandemic. Not all the families in the same cluster before the pandemic were characterized under the same cluster during COVID-19. Interestingly, however, regardless of their pre-pandemic cluster, a high percentage of the families added the appraisal strategy during the pandemic. Positive coping strategies are postulated to mitigate the effects of stress on parenting. In this study, appraisal represented positive coping strategies emphasizing strength to overcome obstacles, reappraising, and making stressors more manageable.

The appraisal theory [10] states that a specific event or stressor influences individual cognitions of an event—termed *appraisal*. Appraisal theory examines the process by which an individual’s subjective interpretation or evaluation of important events or situations elicits emotions. Hence, it is a person’s evaluation of events to determine their safety relative to their place in the environment. Coping ensues after the individual appraises the acute event. An individual uses coping strategies in one of two ways. *Problem-focused coping* focuses on situation-specific goals and allows a sense of mastery and control in working toward attaining those goals. Alternatively, *emotion-focused coping* involves positive reappraisal—cognitively reframing difficult thoughts in a positive manner [20]. This study’s results support the importance of emotion-focused coping in situations over which the individual has limited control (e.g., COVID-19), specifically the importance of positive appraisal in stressful life events.

We found significant interactions between families’ coping-strategy clusters and time for family interaction, parenting, and emotional well-being. These results align with other studies that dealt with families during COVID-19 and described the pandemic’s tremendous consequences on family well-being (e.g., [54, 55]). Our study showed that only families in Cluster 3 reported a decrease in all four measured FQOL aspects. Among families in the other three clusters, FQOL aspects either increased or did not change. These findings highlight the previously mentioned positive impacts of the pandemic and its consequences for families. For example, according to Shek [56], lockdown brought family members more time at home, promoting family cohesion and providing more opportunities for interaction among family members.

Positive emotions, such as comfort, happiness, joy, love, and gratitude, can help maintain and improve mental health. However, given the positive emotions often experienced in interpersonal relationships, the current situation—which requires social distancing and more home time with family members—might explain the positive emotions parents and their children experienced. It may have helped them maintain their well-being, specifically in parenting and family-relations aspects. Comparing coping abilities among families in Cluster 3 with families in the other three clusters again strengthens understanding of the importance of positive appraisal. As presented in the Results section, Cluster 3 describes families that did not view the pandemic positively or as a chance to grow and develop. In this study’s terminology, embracing the challenges and treating them as opportunities [57] translates to positive appraisal and might explain the families’ ability to maintain or enhance FQOL aspects during COVID-19.

### Limitations, implications, and future directions

This study allowed us to investigate how parents cope in a unique situation. When Israel instituted its first lockdown, parents were forced to cope with a previously unknown stressor. Because lockdowns were a tool implemented worldwide to contain the spread of COVID-19, it seemed important to examine their possible effects on families.

Despite this study’s uniqueness and interesting results, it has limitations. The study sample was relatively homogeneous in demographic variables. As with surveys of this type, our sample was restricted to parents with Internet access and willingness to participate. Thus, the results cannot automatically be generalized to other families in Israel. Another limitation of the current study is that parents’ reports of pre-pandemic coping strategies might be prone to recall bias. Thus, changes in coping since the onset of the crisis might be underestimated. Research has suggested insights into retrospective questions during COVID-19: Respondents tend to remember their past as more similar to their present (rather than idealizing the past) when their experience or feeling of interest changed between the time points of interest (past) and data collection (present) [58].

Despite their limitations, this study’s results have important implications. First, emotion-focused coping emerged as a protective factor that affected FQOL positively. Considering their positive effects on well-being and FQOL, emotion-focused coping strategies, specifically positive appraisal, might be a good target for interventions to help parents use more adaptive coping strategies than they had been using. Other implications relate to interactions among family members, their relationships with positive emotions, and their significant effect on well-being. Our research found that being together at home and closely interacting promote family cohesion and strengthen the family’s ability to cope in difficult times. Thus, parents should create more opportunities for family time in day-to-day regular times. We recommend that future studies examine families’ coping profiles relative to other family crises and compare them to this study’s findings. Further, our results regarding the positive effects on FQOL highlight the need for more studies to assess family coping strategies and the potential for positive growth in crises.

Assessments of coping are necessary because results might help therapists identify parents’ needs and design counseling or training programs for stress reduction and coping strategies. Such intervention programs might promote the parents’ ability to adapt their coping strategies and maintain or even enhance aspects of FQOL during crises.

## Conclusion

During the COVID-19 pandemic, it is imperative to understand how families have coped with such a major disaster. Our study captured positive and negative impacts and found coping-strategy differences between before and during the first lockdown. Alongside decreased material well-being, we found no differences—or improved—FQOL in parenting and family relations during the pandemic. The ability to use positive cognitive appraisal was a protective factor against a significant decrease in FQOL during the pandemic.

## Supporting information

S1 Table Family Crisis Oriented Personal Evaluation Scales (F-COPES) items divided into its five scales. (DOCX)

S2 Table Differences in family pandemic-oriented personal evaluation scale factors before and during the COVID-19 pandemic. (DOCX)

S3 Table Interactions between family coping-strategy clusters before and during the COVID-19 pandemic on family quality of life scale factors during the pandemic. (DOCX)
