# Supplementary material for: Coping strategies of families and their relationships with family quality of life during Covid-19 pandemic
Source: PLoS One. 2022 Sep 30;17(9):e0273721. doi: 10.1371/journal.pone.0273721 (PMC9524635; doi:10.1371/journal.pone.0273721)
Supplement: S1 Table — (DOCX) [file pone.0273721.s001.docx]

**Table S1. Family Crisis Oriented Personal Evaluation Scales (F-COPES) items divided into its five scales.**

| **Strategy style** | **No.** | **Item** |
| --- | --- | --- |
| **Acquiring social support** | 1 | Sharing our difficulties with relatives |
|  | 2 | Seeking encouragement and support from friends |
|  | 5 | Seeking advice from relatives (grandparents, etc.) |
|  | 8 | Asking neighbors for favors and assistance |
|  | 14 | Sharing concerns with close friends |
|  | 18 | Doing things with relatives (get-together, dinners, etc.) |
|  | 26 | Sharing problems with neighbors |
| **Reframing** | 3 | Knowing we have the power to solve major problems |
|  | 7 | Knowing we have the strength within our family to solve our problems |
|  | 10 | Facing problems “head-on” and trying to get solutions right away |
|  | 13 | Accepting stressful events as a fact of life |
|  | 17 | Accepting that difficulties occur unexpectedly |
|  | 20 | Believing we can handle our own problems |
|  | 22 | Defining the family problem in a more positive way so that we do not become too discouraged |
| **Seeking spiritual support** | 12 | Attending church services |
|  | 21 | Participating in church activities |
|  | 24 | Seeking advice from a minister |
|  | 27 | Having faith in God |
| **Mobilizing family to acquire and accept help** | 4 | Seeking information and advice from people in other families who faced the same or similar problems |
|  | 6 | Seeking assistance from community agencies and programs designed to help families in our situation |
|  | 9 | Seeking information and advice from the family doctor |
|  | 19 | Seeking professional counseling and help for family difficulties |
| **Passive appraisal** | 11 | Watching television |
|  | 15 | Knowing luck plays a big part in how well we are able to solve family problems |
|  | 23 | Feeling that no matter what we do to prepare, we will have difficulty handling problems |
|  | 25 | Believing that if we wait long enough, the problem will go away |
