# Supplementary material for: Coping strategies of families and their relationships with family quality of life during Covid-19 pandemic
Source: PLoS One. 2022 Sep 30;17(9):e0273721. doi: 10.1371/journal.pone.0273721 (PMC9524635; doi:10.1371/journal.pone.0273721)
Supplement: S2 Table — (DOCX) [file pone.0273721.s002.docx]

**S1 Table**

| **Strategy** | **Before** | | **During** | | ***T* (*df* = 252)** | ***p*** |
| --- | --- | --- | --- | --- | --- | --- |
|  | ***M*** | ***SD*** | ***M*** | ***SD*** |  |  |
| **Acquiring** | 3.00 | 0.72 | 2.92 | 0.67 | 2.67 | < .01 |
| **Reframing** | 3.82 | 0.64 | 3.81 | 0.73 | 0.04 | .97 |
| **Seeking** | 2.69 | 1.08 | 2.56 | 0.96 | 3.05 | < .01 |
| **Mobilizing** | 2.70 | 0.86 | 2.65 | 0.85 | 2.73 | < .01 |
| **Appraisal** | 3.40 | 0.76 | 3.44 | 0.71 | -1.24 | .26 |
| **Total** | 3.20 | 0.43 | 3.14 | 0.44 | 2.45 | .01 |
