# Supplementary material for: Coping strategies of families and their relationships with family quality of life during Covid-19 pandemic
Source: PLoS One. 2022 Sep 30;17(9):e0273721. doi: 10.1371/journal.pone.0273721 (PMC9524635; doi:10.1371/journal.pone.0273721)
Supplement: S3 Table — (DOCX) [file pone.0273721.s003.docx]

**S2 Table**

| **Outcome** | **Family type** | **Before** | | **During** | | ***F* (Interaction) / *t*** | ***p*** | **η²/d** |
| --- | --- | --- | --- | --- | --- | --- | --- | --- |
|  |  | ***M*** | ***SD*** | ***M*** | ***SD*** |  |  |  |
| **Family interaction** |  |  |  |  |  | 18.41 | < .001 | .18 |
|  | **1** | 26.82 | 3.25 | 27.17 | 3.07 | -1.73 | .090 | .20 |
|  | **2** | 26.03 | 3.87 | 26.25 | 4.16 | -0.90 | .370 | .05 |
|  | **3** | 25.82 | 5.00 | 20.94 | 8.78 | 2.96 | .009 | .68 |
|  | **4** | 22.23 | 5.05 | 22.83 | 5.58 | -1.80 | .080 | .11 |
| **Parenting** |  |  |  |  |  | 17.74 | < .001 | .18 |
|  | **1** | 25.52 | 3.72 | 25.66 | 3.88 | -0.84 | .400 | .04 |
|  | **2** | 25.23 | 3.66 | 25.38 | 4.11 | -0.65 | .510 | .04 |
|  | **3** | 26.59 | 3.02 | 22.29 | 8.04 | 2.57 | .020 | .71 |
|  | **4** | 22.20 | 4.35 | 22.59 | 4.61 | -1.93 | .060 | .09 |
| **Emotional well-being** |  |  |  |  |  | 9.58 | < .001 | .10 |
|  | **1** | 16.17 | 2.86 | 15.80 | 3.11 | 2.21 | .300 | .12 |
|  | **2** | 16.37 | 2.95 | 15.92 | 3.51 | 2.09 | .040 | .14 |
|  | **3** | 17.47 | 2.37 | 14.47 | 5.41 | 2.82 | .012 | .72 |
|  | **4** | 13.93 | 3.15 | 13.89 | 3.73 | 0.19 | .850 | .01 |
| **Material well-being** |  |  |  |  |  | 11.45 | < .001 | .12 |
|  | **1** | 22.10 | 3.13 | 21.18 | 3.99 | 3.68 | < .001 | .26 |
|  | **2** | 21.81 | 3.06 | 20.21 | 4.22 | 5.67 | < .001 | .43 |
|  | **3** | 22.53 | 2.43 | 17.47 | 6.34 | 3.78 | .002 | 1.05 |
|  | **4** | 18.93 | 3.81 | 18.05 | 4.18 | 2.82 | .006 | .22 |
